# Macroevolutionary consequences of mast seeding

**DOI:** 10.1098/rstb.2020.0372

**Published:** 2021-12-06

**Authors:** Esther E. Dale, Jessie J. Foest, Andrew Hacket-Pain, Michał Bogdziewicz, Andrew J. Tanentzap

**Affiliations:** ^1^ Manaaki Whenua - Landcare Research, Dunedin, New Zealand; ^2^ Ecosystems and Global Change Group, Department of Plant Sciences, University of Cambridge, Cambridge CB2 3EA, UK; ^3^ Department of Geography and Planning, School of Environmental Sciences, University of Liverpool, Liverpool, UK; ^4^ Department of Systematic Zoology, Faculty of Biology, Adam Mickiewicz University, Ul. Uniwersytetu Poznańskiego 6, Poznań 61-614, Poland; ^5^ INRAE, LESSEM, University Grenoble Alpes, Saint-Martin-d'Heres, France

**Keywords:** seed production, macroevolution, diversification, seed mass, trait evolution

## Abstract

Masting characterizes large, intermittent and highly synchronous seeding events among individual plants and is found throughout the plant Tree of Life (ToL). Although masting can increase plant fitness, little is known about whether it results in evolutionary changes across entire clades, such as by promoting speciation or enhanced trait selection. Here, we tested if masting has macroevolutionary consequences by combining the largest existing dataset of population-level reproductive time series and time-calibrated phylogenetic tree of vascular plants. We found that the coefficient of variation (CV*_p_*) of reproductive output for 307 species covaried with evolutionary history, and more so within clades than expected by random. Speciation rates estimated at the species level were highest at intermediate values of CV*_p_* and regional-scale synchrony (S*_r_*) in seed production, that is, there were unimodal correlations. There was no support for monotonic correlations between either CV*_p_* or S*_r_* and rates of speciation or seed size evolution. These results were robust to different sampling decisions, and we found little bias in our dataset compared with the wider plant ToL. While masting is often adaptive and encompasses a rich diversity of reproductive behaviours, we suggest it may have few consequences beyond the species level.

This article is part of the theme issue ‘The ecology and evolution of synchronized seed production in plants’.

## 1. Introduction

Mast seeding or masting describes synchronous seed production among individual plants and populations that corresponds with large, intermittent reproductive events [1,2]. This adaptation has evolved separately many times across the plant Tree of Life (ToL) [3–5], because it can ultimately increase fitness by conferring economies of scale that reduce the costs of reproduction per surviving offspring [2]. Masting is selected by different pressures [2], such as pollination efficiency [6,7] or predator satiation [8–12]. However, little is known about whether it changes evolutionary processes across species, that is macroevolution, defined by rates of speciation, extinction and phenotypic evolution. The only attempts to address this question have found that some reproductive behaviours associated with masting are evolutionarily conserved across the plant ToL [3–5] or focus specifically in evergreen wet tropical forests [13]. These studies have also considered only some of the ways to measure masting, focusing on temporal autocorrelation [4] and/or temporal variability [3,5], or using a binary classification of masting [13].

**Figure 1.**
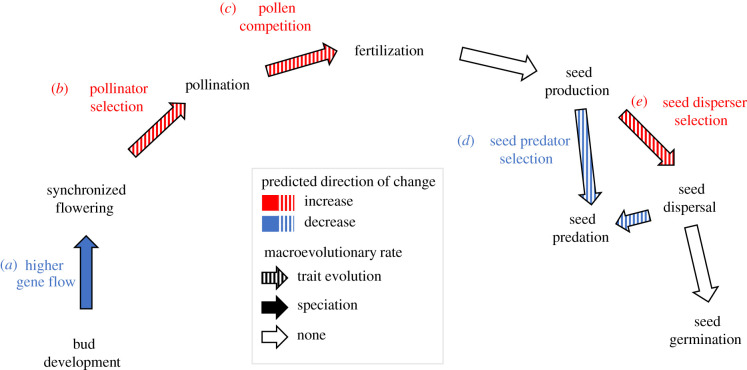
Potential macroevolutionary effects of masting behaviours. Arrows show transitions between reproductive stages in masting species with the type of arrow indicating mechanisms for macroevolutionary effects. Arrow shading indicates a possible influence on a macroevolutionary rate, and colouring indicates the predicted direction of change in that macroevolutionary rate. (Online version in colour.)

Reproductive behaviours associated with masting may influence the macroevolution of species, namely their diversification and trait evolution (figure 1), especially compared to other taxa in the ToL. These behaviours can act as novel innovations if they promote a change in speciation or extinction rates, and thus net diversification rates [14]. Traits can alter speciation rates if they impact adaptation or gene flow directly, such as through changes in pollen placement that reduce gene flow [15]. Traits can also indirectly influence macroevolution, for example, by enabling more environments to be occupied, making allopatric speciation more likely [16]. Extinction can also be influenced by traits, typically via changes in population size, range size or dispersal ability [17]. Two traits that characterize masting behaviours, and thus may influence macroevolution, are the degree of synchrony and temporal variability of reproduction [18].

Synchronization of flowering within and between populations could have macroevolutionary effects through increased pollen competition, higher intensity of selection from pollinators and changes in gene flow. Pollen competition can be more intense in mast years, theoretically allowing adaptations to spread more quickly through the gene pool and hastening phenotypic evolution [19]. More animal pollinators are also attracted to the large floral displays of masting species that exhibit synchronized flowering [20]. This increase in pollinator visitation is likely to intensify pollinator-mediated selection, which could hasten phenotypic evolution in floral traits. Finally, high flowering synchrony at either a population- or regional-scale may dampen speciation rates by promoting gene flow within species.

Large inter-annual variation in seed set and high synchrony in seed production [2,18] can also influence macroevolution through selective pressures from dispersers or predators of seeds. Regional synchrony can influence selection from seed predators, with the direction of that selection depending on the degree of synchrony [12]. For example, in *Pinus pinea*, the directional selection from seed predators on inter-annual variability of seed production had opposite effects for highly synchronized and poorly synchronized plants [12], demonstrating that disruptive selection due to masting is possible. The selective pressures of predators on traits that are not directly involved in masting should effectively be diluted by large temporal variation that causes predators to starve in non-mast years and be satiated in mast years. By contrast, large inter-annual variation in seed production which attracts seed dispersers to fruit displays in mast years is likely to intensify selection on reproductive traits from seed dispersers.

Masting is typically measured in two main ways that can be associated with macroevolutionary change. First, the economies of scale that select for masting require large inter-annual variation in seed set [2,18]. These can be estimated by the population-level coefficient of variation (CV*_p_*) in seed production [5,12]. In species with a large CV*_p_*, seed predators and dispersers may have contrasting outcomes on the selection of phenotypic traits associated with reproduction, e.g. seed size (figure 1*d*,*e*). Seed predators can be choosier in years with high-seed production and dampen phenotypic selection because seeds within a relatively wider trait range can survive. By contrast, traits selected by seed dispersers may evolve more quickly. Seed dispersers can be highly selective about which seeds to disperse in high seed years [21], resulting in a smaller proportion of seeds gaining dispersal benefits, and therefore more intense selection on seed traits than in years with a smaller seed crop.

Second, most economies of scale also require high synchrony in seed production [2,18]. Synchrony is measured between individuals [11,12], but large-scale spatial synchrony between populations over 100s to 1000s km (‘regional synchrony’, S*_r_*) may also be adaptive [22]. Few studies, however, have enough long-term time series located within dispersal distance of each other to calculate S*_r_*. High S*_r_* could strengthen selection on reproductive traits and therefore promote faster trait evolution, such as if pollinators or seed dispersers are attracted to large floral or fruit displays [23,24]. Species with high S*_r_* may also experience relatively higher pollen competition because their pollination efficiency is higher [6], and they can receive pollen from more individuals, thereby promoting faster trait evolution [19]. Conversely, high S*_r_* could weaken selection from seed predators. Seed predators can consume a smaller proportion of seeds before being satiated and therefore exert less selection, when many seeds are available during a short period compared to if the same number of seeds was produced over a longer period. Speciation may also be slower in species with high S*_r_*, which promotes gene flow and therefore limits reproductive isolation. Although the potential selection effects can act in opposite ways, overall there are more mechanisms for promoting faster trait evolution with higher values of both CV*_p_* and S*_r_* through pollinators, seed dispersers and pollen competition (figure 1).

Our aim was to explore whether masting behaviours have wider implications for species and phenotypic diversification beyond their selective advantages for individual fitness [11]. We exploited a new database of 5057 population-level reproductive time series from 682 species—the largest synthesis of reproductive time-series data to date [25]. We first explored the utility of this database for evolutionary inference by comparing biases in the macroevolutionary characteristics of its species with the wider plant ToL. We then tested whether more extreme reproductive behaviours, defined by high values of CV*_p_* or S*_r_*, were correlated and evolutionarily conserved. We used CV*_p_* and the autocorrelation coefficient at a lag of 1 year (AR1) to identify different reproductive strategies and compare their macroevolutionary rates. Using the largest available estimates of species-specific speciation [26] and seed size evolution [27] for vascular plants, we also tested if species with more extreme reproductive behaviours were evolving more quickly in number and seed phenotype.

## 2. Methods

### (a) Reproductive time series

Seed and fruit data were obtained from MASTREE+ [25]. MASTREE+ comprises species-specific time series of annual reproductive effort for perennial plants and includes measures of flower, pollen, fruit and seed production from wild-grown populations (agricultural crops and experimentally manipulated plots were excluded). Data were extracted from the published and grey literature and unpublished datasets. A single time series is considered the set of annual observations described by a unique combination of species, sample location, reproductive variable (e.g. flower, fruit, seed) and measurement unit (e.g. fruit m^−2^).

We subset MASTREE+ to include only time series with observations of fruit, cone or seed production. We restricted our analyses to time series with at least five consecutive years of observations to analyse a larger sample size while maintaining a time series length comparable to previous work [1,4,5,28] and similar in CV*_p_* to longer time series (electronic supplementary material, figure S1). For each time series, we assessed inter-annual variability of reproduction by calculating the coefficient of variation at the population-level (CV*_p_*) [5]. Our analysis was at the species level, so we averaged values for species with multiple independent time series. To estimate the spatial synchrony of reproduction, we calculated pairwise Euclidean distances between the geographical coordinates of all independent time series for each species. We then calculated the Spearman rank correlation coefficient between all pairs of time series within 100 km of each other and all time series generally. Correlation coefficients were averaged at both scales (100 km and globally) to obtain species-level means of spatial synchrony (S*_r_*). We only present results for global synchrony, but those at a 100 km scale were generally consistent, albeit with smaller sample sizes (electronic supplementary material, figure S2 and tables S1–S7).

### (b) Phylogenetic data and macroevolutionary estimates

We analysed two complementary measures of speciation in the plant ToL. We focus on speciation, rather than diversification, because our predictions of potential masting effects on macroevolution involve changes in speciation, not extinction rates. First, we used existing estimates of speciation rates generated for 73 934 vascular plants with Bayesian analysis of macroevolutionary mixtures (BAMM) [26]. BAMM models heterogeneity in speciation and extinction through time and across lineages and can account for non-random incomplete taxon sampling [29]. It has been extensively validated for estimating speciation rates [30,31]. The BAMM speciation rate estimates we obtained here were generated using the most comprehensive time-calibrated phylogenetic tree of seed plants presently available, i.e. GBOTB in Smith & Brown [32]. Second, we estimated speciation rates with the diversification rate metric (DR) [33] using the GBOTB phylogeny. DR considers only branch lengths and splitting events and does not accommodate incomplete taxon sampling [33], so we focus on BAMM-derived rates in the main text. We used the GBOTB phylogeny for all subsequent analyses that required a phylogenetic tree.

We also tested if masting was associated with the rate of evolutionary change in an important reproductive trait, seed size, which should be under selection from both seed dispersers and predators [34,35]. We collated existing estimates of the rate of seed size evolution across the plant ToL generated for 13 579 angiosperms [27]. Briefly, seed mass data were obtained from the Royal Botanic Gardens Kew Seed Information Database (http://data.kew.org/sid/) and intersected with a time-calibrated phylogenetic tree for land plants generated with publicly available sequences for seven gene regions [36]. Speciation rates estimated with BAMM for this phylogeny have been shown to be correlated with those from the larger GBOTB tree [26]. BAMM was then used to model rates of seed size evolution (see [27] for full details). The rate of seed size evolution was assumed to follow a different Brownian motion process within each clade of species estimated by BAMM to share macroevolutionary dynamics [37].

There were 307 woody and herbaceous species (65 families) in both MASTREE+ and the GBOTB phylogeny from 45 countries (electronic supplementary material, figure S3). Of these species, all had CV*_p_* and DR estimates, 105 had a global synchrony estimate and 274 and 149 had BAMM speciation and seed size evolution estimates, respectively. This subset was generated from 10 687 records in 870 time series (median time series length: 10 years, range: 5 to 62 years), with the highest density of time series in North America and Europe (electronic supplementary material, figure S3). Pinaceae, Fagaceae and Fabaceae were the best-represented families in terms of species, with 47, 37 and 22 species, respectively. Oak, *Quercus*, was the most represented genus with 34 species. We resolved all taxonomic names to the species level in the reproductive time-series data, phylogeny and BAMM objects using The Plant List [38] in R v. 4.0.3 [39]. Full details are given in the electronic supplementary material, Methods.

### (c) Clustering reproductive strategies

Different reproductive behaviours can be characterized as masting. Grouping these behaviours into a single ‘masting’ category may obscure correlations with macroevolutionary rates if different groups are associated with speciation and trait evolution in opposing ways. To complement our species-level analyses and test for macroevolutionary differences between contrasting reproductive strategies, we divided taxa into clusters based on their CV*_p_* and the autocorrelation coefficient at a lag of 1-year (AR1) values. Although there is no clear indication of how AR1 might directly influence macroevolution, it could do so along with CV*_p_* by characterizing different masting strategies [18]. A Gower's distance dissimilarity matrix was generated, using the *daisy* function in the ‘cluster’ R package [40], which was k-means clustered with the *kmeans* function from the ‘stats’ package. We performed this cluster analysis with 2–12 clusters and, based on the elbow method [41] of mean sum of squares (see the electronic supplementary material, figure S4), we selected five clusters to use for subsequent analyses.

### (d) Biases in masting data

MASTREE+ has positive biases to long-lived, woody species from temperate latitudinal regions, so we also tested if it had macroevolutionary biases or was generally representative of the plant ToL. We did so by comparing the speciation rates (both BAMM and DR) and rate of seed mass evolution, of our 307 study species to all other seed plants in the GBOTB phylogeny (*n* = 79 574). BAMM speciation rate and seed mass evolution were compared between groups using structured rate permutations on phylogenies (STRAPP) via the *traitDependentBAMM* function in the ‘BAMMtools’ package [42] in R. STRAPP calculates the association between macroevolutionary rates and a binary variable of occurrence in the reproductive time-series data using a Mann–Whitney U-test. The observed U-statistic is then compared to a distribution of null values to estimate statistical significance. These null correlations were generated by permuting the macroevolutionary rates across the tips of the phylogeny 1000 times while maintaining the position of estimated rate shifts in the phylogeny. For DR, we compared MASTREE+ and GBOTB species by fitting a binomial phylogenetic generalized linear model to a binary variable of whether species occurred in the reproductive time-series dataset (see electronic supplementary material, Methods).

### (e) Hypothesis testing

We tested if species-level masting behaviours, defined by CV*_p_* and S*_r_*, were evolutionarily conserved using Pagel's *λ* [43] and the *K* statistic [44]. *λ* estimates how trait similarity correlates with phylogenetic similarity, indicating the degree to which trait values reflect shared evolutionary history. The *K* statistic, by contrast, compares trait variation within clades to variation among clades, so can detect phylogenetic clustering or overdispersion [43,44]. With each masting metric as a response variable, we estimated Pagel's *λ* using phylogenetic generalized least squares (PGLS) with the *gls* function in the ‘nlme’ package and a correlation structure generated for the pruned GBOTB phylogeny using the *corPagel* function in the R package ‘ape’. We constrained *λ* to between 0 and 1. For the species with standard errors for the masting metrics (i.e. across replicate time series), we also quantified *λ* incorporating the errors into the PGLS by weighting observations with the inverse variances [45]. We tested the statistical significance of *λ* values using the *anova* function in the ‘nlme’ package to compare each PGLS to an equivalent null model fitted without the correlation structure. We estimated the *K* statistic for each species using the phylogeny pruned from GBOTB and the *phylosig* function in the ‘phytools’ package in R. We tested the statistical significance of each *K* statistic with *phylosig*, which compares the estimated *K* value to a null distribution generated by randomly shuffling tips in the phylogeny 1000 times. These phylogenetic signal calculations were repeated with and without sampling error. Masting metrics were considered phylogenetically clustered if less than 2.5% of the *K* values from the randomized distribution exceeded the observed *K*, and over-dispersed if less than 2.5% of the null *K* values were smaller than the observed *K*.

We tested if reproductive behaviours defined by CV*_p_* and S*_r_* were phylogenetically correlated (i.e. coevolving) or evolving independently of each other. We fitted a PGLS regression between the two metrics with the *pgls* function in the ‘caper’ R package. We used a pruned GBOTB phylogeny and scaled errors along with the phylogeny by estimating Pagel's *λ*, as is standard practice [46].

To examine the macroevolutionary consequences of masting, we tested whether CV*_p_* and S*_r_* were associated with BAMM and DR speciation rates, and rates of seed mass evolution. For BAMM speciation rates and rates of seed mass evolution, we used STRAPP described above but with the Spearman rank correlation coefficient. For DR, we used the equal-splits with simulation test (electronic supplementary material, Methods). We also used quantitative state speciation and extinction (QuaSSE) models to test the association between speciation rate and CV*_p_* and S*_r_* [47]. We fitted maximum-likelihood models with constant, linear, sigmoidal or modal speciation functions to each masting metric and constant extinction using the ‘diversitree’ R package. We identified the best-supported speciation model for each metric with the Akaike information criterion (AIC).

We also tested whether macroevolutionary rates varied among groups of species with different reproductive behaviours that would have been masked in the species-level analysis. The macroevolutionary differences between clusters of species in the reproductive time-series data were compared using several examined and concealed state-dependent speciation and extinction (SecSSE) models for BAMM speciation rates, PGLS for DR speciation rates, and STRAPP for the BAMM-estimated speciation rate and the rate of seed mass evolution. We used the ‘SecSSE’ R package [48] to fit an examined-trait-dependent (ETD) model where speciation rates vary among different reproductive clusters. To assess if the ETD model was supported by our data, we compared it to a null model with constant speciation rates for all species (CR) and a concealed-trait-dependent (CTD) model where speciation rates varied independently of the pre-defined reproductive clusters. For the CTD model, we instead let speciation rates vary among five different but unknown clusters, termed ‘concealed states’, as recommended by [49]. Extinction rates and transition rates between states were fixed across all states for all three models. Starting values were generated using a simple birth-death model with the *bd_ML* function in the ‘DDD’ R package [50], and we reran the models with values doubled and halved to avoid only finding local maxima. We identified the best model by comparing AIC weights (AIC_w_) [51]. The PGLS model was fitted as described for metric correlations, with the GBOTB phylogeny and DR speciation values as the response and the cluster each species belonged to as an explanatory factor. The STRAPP analysis tested the correlation between BAMM speciation rate or seed mass evolution and the cluster each species belonged to using the Kruskal–Wallis method and 1000 permutations. We also examined the differences in spatial synchrony and absolute latitude between masting clusters with PGLS. Synchrony was negatively skewed so was exponentially transformed beforehand. When fitting the PGLS we co-estimated Pagel's *λ* in the same way as described above and the *p*-values were not corrected for multiple tests.

To test the robustness of the results to our filtering decisions, we repeated all the analyses with different data subsets. We repeated the analyses with reproductive time series of at least 3, 4 and 6 years of consecutive observations to test the effect of the 5-year filtering decision. Similarly, we tested the effect of unit type by separately analysing datasets of only mass-based, counts-based, per-area or per-individual units. All of the code for performing the analyses can be found on Zenodo [52].

## 3. Results

### (a) Macroevolutionary biases

Macroevolutionary rates were generally representative of those across the wider plant ToL. Both BAMM-estimated rates of speciation and seed mass change were comparable between MASTREE+ and the wider plant ToL (electronic supplementary material, figure S5a,b). The DR metric of recent speciation was only a mean of 7% lower (0.85 species Ma^−1^) in MASTREE+ species than in the wider GBOTB phylogeny (electronic supplementary material, figure S5c).

**Figure 2.**
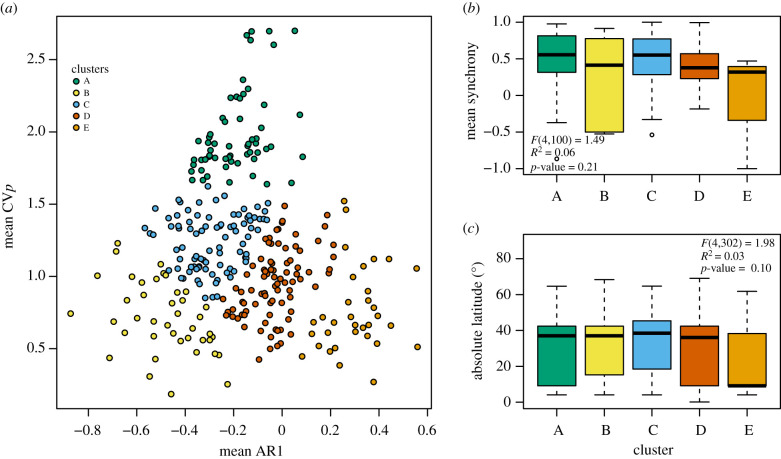
Different reproductive strategies across the plant ToL. (*a*) Species cluster into five groups based on their population-level coefficient of variation (CV*_p_*) and lag-1 autocorrelation (AR1). Metrics were averaged across replicate time series for 307 species. Clusters did not vary in mean (*b*) spatial synchrony globally and (*c*) absolute latitude. PGLS models testing the effect of clusters are shown inset for (*b*) and (*c*). (Online version in colour.)

We further related different reproductive strategies to macroevolutionary characteristics by grouping MASTREE+ species into clusters based on their CV*_p_* and AR1 values. We found that five clusters best described the data (electronic supplementary material, figure S4; figure 2*a*). Although these clusters did not exhibit statistically significant differences in S*_r_* and latitude, their values helped us interpret the corresponding reproductive strategies (figure 2*b*,*c*). For example, cluster A, defined by high CV*_p_*, consisted of species with extreme inter-annual variation in seed crop. By contrast, cluster E, defined by low CV*_p_* and positive AR1, indicated masting where seed production is similar among years. The remaining clusters B, C and D generally differed in AR1, indicating that they may reflect different resource use strategies or environments that cause different sequences of low- and high-seed years without necessarily leading to large absolute differences between years.

### (b) Phylogenetic patterns

**Figure 3.**
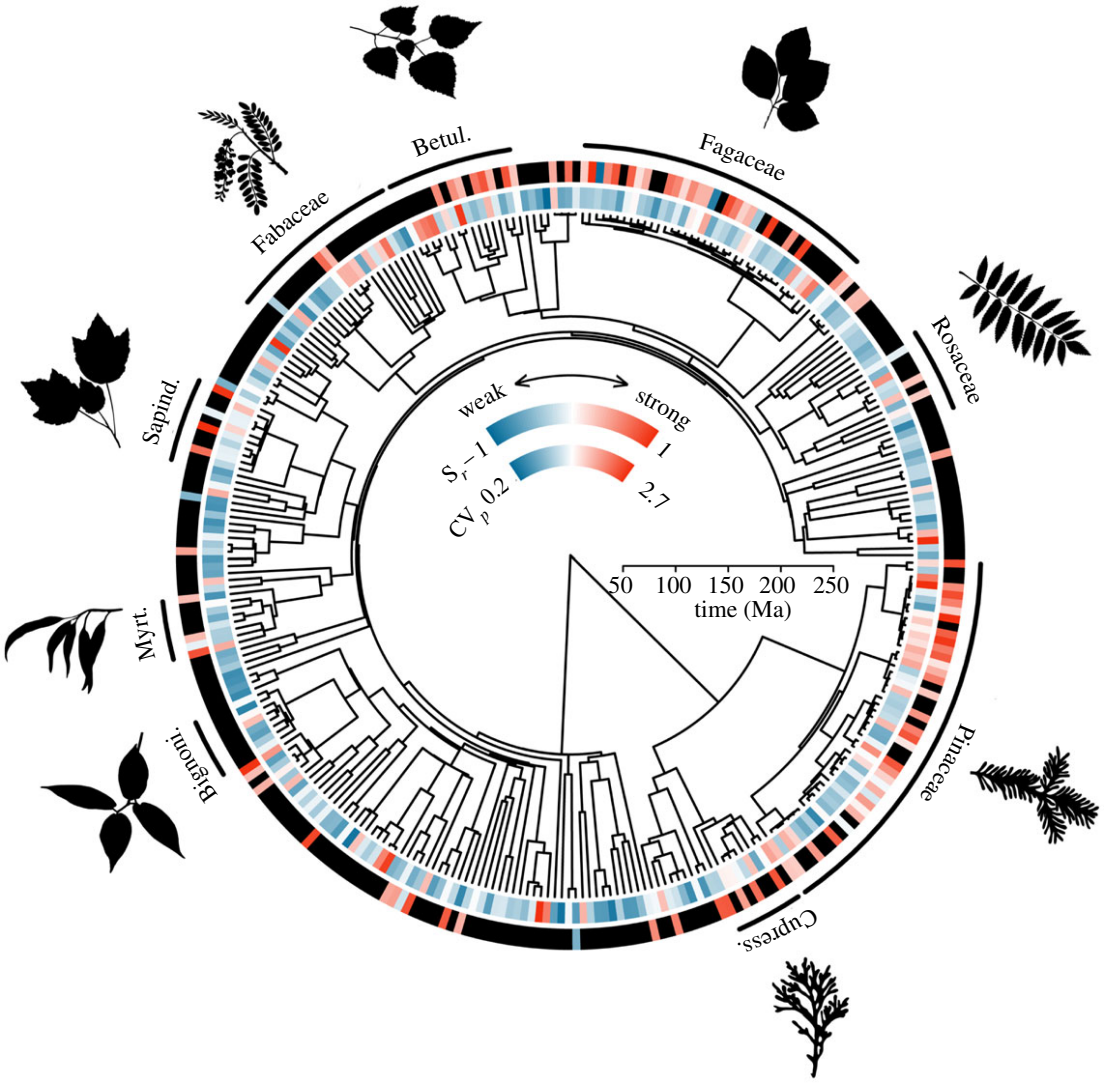
Masting behaviour varies across the plant ToL. Time-calibrated phylogeny from Smith & Brown [27] displaying mean species spatial synchrony (S*_r_*) and coefficient of variation in population-level reproduction (CV*_p_*) (*n* = 870 time series from 307 species for CV*_p_*, *n* = 652 time series from 105 species for S*_r_*). Values range from blue, indicating weak masting behaviour, to red, indicating strong masting behaviour, with black indicating missing values (*S_r_* only). Plant families with at least eight species included in the study are shown. Plant silhouettes are from phylopic.org (Ferran Sayol and Mattia Menchetta) released under a Public Domain Dedication 1.0 licence. (Online version in colour.)

Masting behaviour varied across the plant ToL. CV*_p_* showed a phylogenetic signal (*λ* = 0.35; table 1), implying that values tend to be similar in related species owing to their shared evolutionary history. However, there was large variation within clades in all masting metrics (*K* < 0.05), with CV*_p_* more over-dispersed than expected, indicating divergence in reproductive strategies within clades (table 1). No statistically significant phylogenetic signal was detected for S*_r_* (table 1). The masting metrics were not phylogenetically correlated (electronic supplementary material, table S8), indicating that strong masting behaviour does not require CV*_p_* and S*_r_* to be coordinated per se (figure 3). Masting behaviours could therefore be found across the phylogeny, having arisen frequently in different ways in different parts of the plant ToL (figure 3).

**Table 1.** Phylogenetic signal in masting metrics across vascular plants. (Pagel's *λ* and Blomberg's *K* statistic tested for phylogenetic signal in species-level means of the coefficient of variation in population-level reproduction (CV*_p_*) and global spatial synchrony (S*_r_*). *λ* tests for phylogenetic correlation between species based on their shared evolutionary history. Corresponding *p*-values were calculated from comparing generalized least squares models with and without a Brownian correlation structure scaled by an estimated *λ*. *p*-values for the *K* statistic test for phylogenetic clustering and overdispersion were derived using a randomization test. Italicized *p*-values are considered statistically significant if less than 0.05 for *λ* and less than 0.025 for *K*. *n* is the number of species.)

			Blomberg's *K* statistic			
	Pagel's *λ*			*p*-value		
masting metric	*λ*	*p*-value	*K*	clustering	overdispersion	*n*
mean CV*_p_*	*0.35*	*0.01*	*0.01*	0.98	*0.02*	307
mean S*_r_*	0.00	0.84	0.03	0.35	0.66	105

### (c) Evolutionary consequences of masting

**Figure 4.**
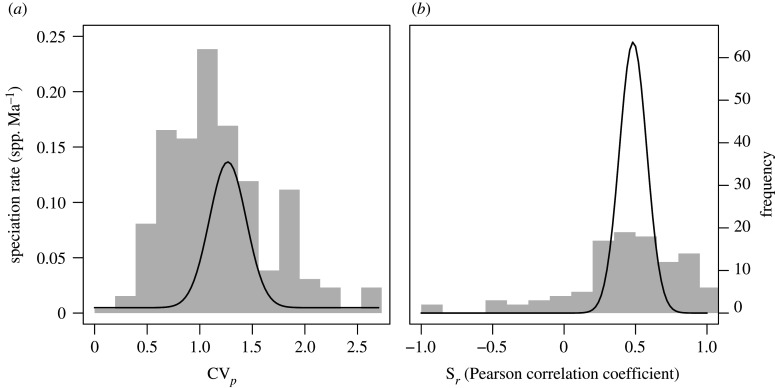
Speciation is highest with intermediate trait values. Modal QuaSSE models relating speciation rate to (*a*) CV*_p_* and (*b*) S*_r_*. Black lines show the modelled fits, and grey bars show the frequency of species.

**Figure 5.**
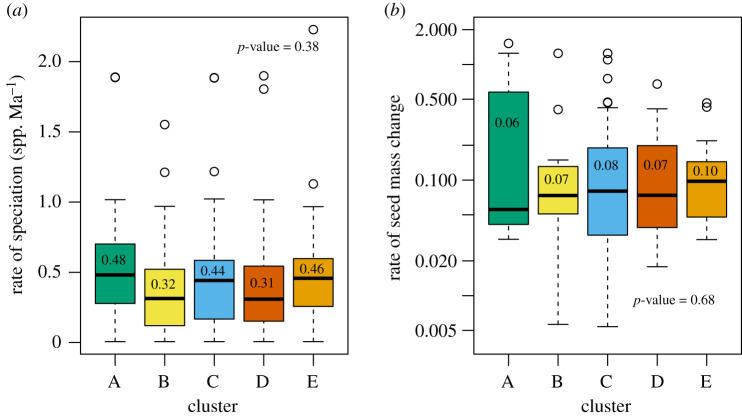
Different reproductive strategies are not associated with different macroevolutionary characteristics. We calculated (*a*) rate of speciation and (*b*) rate of seed mass evolution. The bold horizontal lines show the median, boxes show interquartile range, whiskers are 1.5 times the interquartile range and points are values outside that range. Letters and colours indicate different reproductive strategies defined using a cluster analysis on AR1 and CV*_p_* (figure 2*a*). Characteristics were compared among clusters using STRAPP, with *p*-values and estimated medians of species-specific rates for each cluster shown on each plot. The rate of seed mass change is unitless. (Online version in colour.)

Masting behaviour was typically not associated with macroevolutionary rates, even when clustered into the different reproductive strategies. There were no statistically significant monotonic correlations between either of the masting metrics and the BAMM-estimated speciation rate or the rate of seed mass evolution, and these correlation coefficients were generally weak (table 2). However, when non-linear associations were included, we found speciation estimated by QuaSSE was highest at intermediate trait values for both masting metrics (figure 4; electronic supplementary material, table S9). There were also no differences in BAMM speciation rate or the rate of seed mass evolution among different reproductive strategies (figure 5). Instead, speciation rates were estimated to vary independently of the pre-defined reproductive strategies by SecSSE (AIC_w_ ∼ 1). Speciation rates varied across five groups that were unrelated to the reproductive strategies with rates ranging from 0.05 to 0.26 spp. Ma^−1^, except for one group that had an estimated rate of 0.72 spp. Ma^−1^. All our findings were robust to the speciation rate (BAMM versus DR), type of units used in analyses and the minimum duration of time series (electronic supplementary material, figure S2 and tables S1–S7).

**Table 2.** Masting metrics are not associated with macroevolutionary rates. (BAMM-estimated speciation and seed size evolution rates were tested using STRAPP. Correlation coefficients were Spearman's rank coefficient.)

masting metric	evolutionary rate	correlation coefficient	*p*-value	*n*
CV*_p_*	speciation	0.11	0.18	274
CV*_p_*	seed mass evolution	0.04	0.60	164
S*_r_*	speciation	0.06	0.51	96
S*_r_*	seed mass evolution	−0.13	0.30	50

## 4. Discussion

Mast seeding is observed in many species [3–5,11,12], but here we found that its fitness benefits translate into few macroevolutionary changes. Species with masting-like reproduction were dispersed across the plant ToL and not clustered in clades, consistent with previous reports of variation in masting among close relatives (e.g. *Quercus*, [53]). Different attributes of long-term seed production (CV*_p_* and S*_r_*) appeared to be evolving independently (electronic supplementary material, table S8). We also detected no association between masting metrics and the rate of seed size evolution, a key trait involved in reproduction. This lack of association can arise if masting behaviours cause opposing macroevolutionary effects within species and/or in different species. Within species, there may be varied selection effects, such as from both seed predators and seed dispersers (figure 1*d*,*e*), which together may result in minimal trait change. For example, stabilizing selection on seed size may arise if both larger seeds are more apparent to predators, particularly in forests that dominate in our dataset [54], and smaller seeds experience more interspecific competition post-dispersal [55]. Alternatively, the same type of selection may occur in opposing ways within species [12,18]. For example in *Quercus ilex*, pre-dispersal predation by weevils should select for large acorns [56], whereas post-dispersal predation by mammals selects for smaller seeds [34]. Likewise, there may be opposing effects in different species, because of their diversity of predators, pollination methods and seed dispersal mechanisms [57,58]. A combination of different types of selection in different species, such as stronger selection from pollinators in animal pollinated species, but a weaker selection from seed predators in animal-dispersed species, may obscure association between rates of trait evolution and masting metrics. Negative results may also have arisen because the fitness benefits associated with different masting metrics vary widely. For example, high CV*_p_* and high S*_r_* may help satiate predators that are highly abundant [11,12], whereas plants with sparse predators may benefit only from high S*_r_* that concentrates flowering effort at one time point to improve pollination [6,59]. These differences in reproductive strategies can also act independently of masting dynamics, such as through long-distance gene flow, common in many of the wind-pollinated tree species in our analysis [60], which can slow macroevolutionary change. Finally, the speciation effects of masting may depend on other reproductive traits that we have not examined, as macroevolutionary rates varied among groups of species but unrelated to the five different strategies defined by CV*_p_* and AR1.

Speciation was highest with intermediate values of both CV*_p_* and S*_r_*. This type of association between synchrony and speciation rates may be caused by opposing effects of S*_r_* on gene flow. We predicted that gene flow would be higher with greater synchrony, but variability in synchrony may promote reproductive isolation in some species, resulting in the highest speciation with intermediate S*_r_* values. If S*_r_* becomes more variable, multiple synchronized groups could arise that are inherently out-of-sync with each other [61]. Consequently, we would expect high gene flow within groups with the same flowering time and low gene flow between groups with different flowering time, which could promote reproductive isolation and faster speciation. Differences in the timing of flowering arising would then promote non-random mating [62,63]. Disruptive selection on timing of flowering could even result in sympatric speciation through temporal reproductive isolation because it maintains assortative mating within groups that flower at different periods, as pollination can only occur between those individuals that flower concurrently [63]. Close relatives that are out-of-sync occur in the bamboos [64] and coexisting oak species that exhibit reproductive cycles of different frequency [65], resulting in masting of a different periodicity in each species, though these differences could have arisen during or after speciation.

A similar process of opposing effects on speciation may operate for CV*_p_*. Species with a high CV*_p_* may have low speciation owing to large floral displays or seed crops attracting dispersers and pollinators [23,24] or increasing effectiveness of wind pollination [66] that could increase gene flow in mast years. Additionally, high CV*_p_* may limit speciation via coevolution with pollinators or seed dispersers because variable seed or flower availability would make specialization difficult. Perhaps there is low speciation with low CV*_p_* owing to less intraspecific seedling competition [67] slowing disruptive selection and therefore speciation rates. The potential for disruptive selection on the synchrony and variability of flowering remains to be examined in masting species.

We advanced previous attempts to explore the evolutionary history of masting by considering spatial synchrony alongside temporal variability, and linking reproductive behaviour to both speciation and trait diversification. Synchrony is important for reconstructing the evolutionary history of masting because it should be strongly involved in reproductive isolation and phenotypic selection. However, its effects can be difficult to detect in large comparative studies like ours because of variation in the life history, diet, mobility, generation time and dietary specialization of predators [12], resulting in no clear generalities. Alternatively, masting can arise because plants are simply matching inter-annual variation in weather that limit the resources required for seed production, or are responding to cues that select for synchrony within populations [2]. As these weather cues are synchronized spatially owing to large-scale climate patterns, synchrony, especially at the 100+ km scale we measured here, may be more an emergent property of this process rather than a direct benefit to fitness [68,69]. Synchrony may have also been unimportant for macroevolution in our study because we calculated it over larger distances (greater than or equal to 100 km) than those at which populations interbreed and experience reproductive isolation. Even with our large dataset, we lacked sufficient replicate time series to calculate S*_r_* at smaller spatial scales. Future studies could overcome these challenges by testing if spatial synchrony promotes genotypic differentiation between populations within species where the economies of scales from predator satiation are greatest.

A further explanation for finding few statistically significant effects in our study is that our methods are sensitive to sample size. STRAPP, for example, may require many hundreds more species to detect statistically significant correlations between traits and speciation rates [70]. BiSSE, which is similar to QuaSSE and SecSSE, requires at least 300 species for reliable results [71]. Related, these associations may occur only for some masting behaviours. Our clustering of species into different reproductive strategies therefore returns to an important question of how masting is itself defined. By finding that masting is an emergent property associated with different reproductive behaviours (CV*_p_*, S*_r_*) that evolve independently from each other, our results are consistent with general theory predicting that diverse, species-specific selective pressures will create a diversity of masting behaviours [18].

The evolution of masting has long been of interest to researchers [72], and efforts to compile large reproductive time-series datasets have enabled important insights, such as the phylogenetic association between masting and nutrient imbalance [4]. Nevertheless, relatively little is known about the wider macroevolutionary consequences of masting. Here, we provide the first steps to address this gap, demonstrating the value of newly available reproductive time-series datasets to investigate the macroevolution of masting [25]. Future work should consider whether the macroevolutionary consequences of masting extend to the evolution of floral morphology and other reproductive traits, as well as non-reproductive aspects of the niche, such as geographical distribution or climate niche. Given the variety of reproductive behaviours and varied combinations of these behaviours that encompass masting, future attempts to understand the evolution of plant reproductive patterns must therefore focus on understanding their complexities and examining how these interact to affect evolutionary change.

## Data accessibility

The scripts for running the analyses can be found in a public repository: https://doi.org/10.5281/zenodo.5093128. A separate publication describing the MASTREE+ dataset is in preparation and the dataset can be accessed via an online data explorer: https://mastreeplus.shinyapps.io/mastreeplus/. The GBOTB phylogeny is available as an electronic supplementary material from Smith & Brown [32] (https://doi.org/10.1002/ajb2.1019). The BAMM objects are from Igea & Tanentzap [26] (https://github.com/javierigea/LDGplants_rates) and Igea *et al*. [27] (https://github.com/javierigea/seed_size).
